# MASCC/ISOO Clinical Practice Statement: imaging and clinical laboratory tests in the diagnosis and management of medication-related osteonecrosis of the jaw

**DOI:** 10.1007/s00520-025-09809-8

**Published:** 2025-09-13

**Authors:** Noam Yarom, Ragda Abdalla-Aslan, Cesar Migliorati, Elena Livshits, Mohammed Amir Rais, Wonse Park, Eduardo R. Fregnani, Kivanc Bektas-Kayhan, Joel Epstein, Sharon Elad

**Affiliations:** 1 Oral Medicine Unit, Sheba Medical Center, Tel Hashomer, 5265601 Israel; 2 The Maurice and Gabriela Goldschleger School of Dental Medicine, Gray Faculty of Medical and Health Sciences, Tel Aviv University, Tel Aviv, Israel; 3 The Department of Oral and Maxillofacial Surgery, Rambam Health Care Campus, Haifa, Israel; 4 Ruth and Bruce Rappaport Faculty of Medicine, Technion Israel Institute of Technology, Haifa, Israel; 5 Oral and Maxillofacial Diagnostic Sciences, Oral Medicine, University of Florida College of Dentistry, Gainesville, FL USA; 6 Oral & Maxillofacial Surgery, Massachusetts General Hospital, Boston, MA USA; 7 Department of Advanced General Dentistry, Yonsei University College of Dentistry, Seoul, Korea; 8 Centro de Oncologia Molecular, Instituto de Ensino e Pesquisa, Hospital Sírio-Libanês, São Paulo, SP Brazil; 9 Department of Oral and Maxillofacial Surgery, İstanbul University Faculty of Dentistry, Istanbul, Turkey; 10 Dental Oncology Services, City of Hope National Medical Center, Duarte, CA USA; 11 Cedars Sinai Health System, Los Angeles, CA USA; 12 Oral Medicine, Eastman Institute for Oral Health, University of Rochester Medical Center, Rochester, NY USA

**Keywords:** Osteonecrosis of the jaw, Bisphosphonates, Bone-modifying agents, Bone turnover markers, Denosumab, Imaging, Radiographs, Blood tests

## Abstract

**Purpose:**

A MASCC/ISOO Clinical Practice Statement (CPS) is aimed at generating a concise resource for clinicians that concentrates practical information needed for the management of oral complications of cancer patients. This CPS focuses on the use of imaging and clinical laboratory tests for the diagnosis, staging, monitoring, treatment decision, and prediction of medication-related osteonecrosis of the jaw (MRONJ) in cancer patients.

**Methods:**

This CPS was developed based on a critical evaluation of the literature followed by a structured discussion of a group of leading experts, members of the Oral Care Study Group (OCSG) of MASCC/ISOO. The information is presented in the form of succinct bullets and tables to generate a short manual about the best standard of care.

**Results:**

Radiographs, cone beam computerized tomography (CT), conventional CT, magnetic resonance imaging (MRI), and nuclear imaging are often utilized in patients with MRONJ. The CPS describes the considerations for selecting each imaging modality. Laboratory workup in patients with MRONJ is often derived by comorbidities, with immune status and bleeding tendency being the key considerations.

**Conclusion:**

Imaging and lab tests have an important role in the diagnosis and management of MRONJ. The imaging modality and specific laboratory tests should be tailored to the patient’s needs.

**Supplementary Information:**

The online version contains supplementary material available at 10.1007/s00520-025-09809-8.

## Introduction

Osteonecrosis of the jaw (ONJ) is a well-recognized complication in cancer patients, and it is associated with bone-modifying agents (BMA), mainly bisphosphonates and denosumab. It has also been reported in association with other medications with a lower prevalence [1, 2]. Accordingly, the American Association of Oral and Maxillofacial Surgeons (AAOMS) coined the term medication-related ONJ (MRONJ) [3].

In cancer patients, BMA are indicated primarily for patients with bone metastases and multiple myeloma to reduce skeletal-related events such as vertebral fractures, spinal cord compression, and hypercalcemia of malignancy. Additionally, BMA have been suggested as an adjuvant therapy in moderate- or low-dose regimens for breast cancer patients [4, 5]. Cancer patients who develop therapy-related osteoporosis may be prescribed relatively low-dose regimens of BMA.

According to widely accepted guidelines, the diagnosis of MRONJ can be made based on its clinical presentation alone [3, 6]. However, imaging studies and laboratory tests are important in various aspects of MRONJ management. The value of imaging was highlighted in the latest Italian guidelines paper [7]. The role of laboratory tests in MRONJ has not been incorporated into formal guidelines or society position papers.

A joint guidelines paper for the prevention and management of MRONJ in cancer patients was published by the Multinational Association of Supportive Care in Cancer (MASCC), the International Society of Oral Oncology (ISOO), and the American Society of Clinical Oncology (ASCO) [6]. The utilization of adjunct tests was not discussed. Therefore, a working group of the Oral Care Study Group (OCSG) of MASCC/ISOO was established to compose an expert-opinion Clinical Practice Statement (CPS) to provide a concise summary about the use of imaging and laboratory tests in the management of MRONJ in cancer patients. This CPS does not refer to the use of imaging as part of the routine dental care that is recommended for all patients treated with BMA.

Imaging and laboratory tests should be interpreted in conjunction with the information about the patient’s medical history, use of medications, and clinical signs and symptoms. Therefore, this CPS complements the guidelines papers referenced above and is not to be used alone in the diagnosis and management of MRONJ patients.

## Objective

To outline the utility of imaging studies and laboratory tests in managing MRONJ in cancer patients through the entire spectrum of patient care: diagnosis, differential diagnosis, staging, monitoring, treatment decisions, and prediction.

## Methods

This CPS is a composite of expert opinion and a high-quality review of the literature. PubMed was searched for data pertinent to MRONJ up to November 2024. The CPS was discussed by a multi-disciplinary Oral Care Study Group (OCSG) working group, experts on MRONJ, and then reviewed by two independent boards: the ISOO Advisory Board and the MASCC Guidelines Committee. The Statement follows the MASCC/ISOO Guidelines Policy.

## Clinical relevance and practical considerations

The considerations for each of the two assay types—imaging and laboratory workup—will be described below according to the pertinent aspects of care: diagnosis, differential diagnosis, staging, monitoring, treatment decisions, and prediction.

### Imaging studies—diagnosis

- Generally, depending on the clinical findings, imaging is unnecessary for the diagnosis of MRONJ; nevertheless, in some cases, it may be critical in the diagnosis and assessment. Often, there is a correlation between the clinical presentation and the radiologic findings; however, there may be cases in which the clinical presentation is non-specific or limited [3, 8]. In such cases, imaging may assist with the diagnosis which then enables prompt management. Additionally, imaging may assist in determining the extent of the necrotic bone and its relationship to adjacent anatomic structures, which is commonly larger than that suggested on clinical grounds alone.
- There is no consensus on the appropriate imaging modalities for each clinical setting, and each option presents its own advantages and disadvantages (Table 1). Clinicians should select appropriate imaging tailored for each individual case.

**Table 1:** Advantages and disadvantages of common imaging modalities for MRONJ

	Advantages	Disadvantages
Peri-apical X-rays	• Presents detailed imaging and high resolution of the involved area compared to Panoramic view • Presents relationship to adjacent dental structures • No metallic artifacts • Relatively inexpensive • Low radiation dose • Commonly available in practice	• Presents a small field • 2D view
Panoramic X-rays	• Presents a large field of view (suitable for large MRONJ lesions) • Demonstrates proximity to anatomic structures • Relatively low radiation dose compared to CBCT and CT	• Compromised view of the anterior jaw area • Overlap of structures and ghost images • Low resolution compared to PA view • 2D view • Low sensitivity in detecting MRONJ compared to CBCT
CBCT	• 3D view • Enables imaging of a limited field of view • Low radiation dose compared to CT* • Less metallic artifacts compared to CT* • Higher resolution compared to CT*	• No soft tissue details • High image noise • Tendency to underestimate extent of lesion
CT	• 3D view • Presents both soft and hard tissues	• Often high radiation dose • Metallic artifacts
MRI	• Best technique for soft tissue imaging • Presents very early changes in bone marrow	• Relatively expensive • Lower availability compared to CT • Limited bony lesion details compared to CBCT and CT • Metallic artifacts
Nuclear imaging	• Performed routinely as part of the metastatic disease monitoring; as such, it may provide an initial indication for a jaw pathology • High sensitivity in detecting MRONJ	• Non-specific uptake (MRONJ vs. dental inflammation vs. metastasis) • Low resolution • Tendency to overestimate extent of lesion

*2D* bi-dimensional, *3D *Ttri-dimensional, *CBCT* cone-beam computed tomography, *CT* computed tomography, multi-detector computed tomography, *MRI* magnetic resonance imaging; *in selected protocols
- Imaging methods utilizing X-rays range from those with the lowest radiation exposure (plain radiography) to the highest radiation exposure (multi-detector computerized tomography; CT). Generally, there are two main selection principles to follow:
  - ○ Justification principle: imaging should only be used if the result may change the diagnosis and/or treatment plan either at baseline or during long-term follow-up.
  - ○ Optimization principle: the radiation dose should be *A*s *L*ow *As D*iagnostically *A*pplicable, being *I*ndication-oriented and *P*atient-specific (ALADA-IP).
- The common presentation of MRONJ in various imaging modalities is provided in Tables 2 and 3.

**Table 2:** Imaging findings associated with BMA and MRONJ — plain radiography

Imaging modality	Imaging features that may be observed
Periapical X-rays [9]	▪ Dental-periodontal related structures: o Thickening of lamina dura o Widened periodontal ligament o Non-healing extraction site (empty socket) ▪ Jawbone: o Radiopaque and/or radiolucent bony lesion o Tends to have poorly defined borders
Panoramic X-rays [10, 11]	In addition to imaging features observed in periapical X-rays: ▪ Jawbone: o Regional or diffused osteosclerosis ▪ More prominent in the alveolar process compared to body of mandible ▪ May extend beyond the site of sequestrum o Sequestrum o Thickening of cortical structures: oblique ridge, lower mandibular border o Prominent mandibular canal o Periosteal reaction (usually in the form of proliferative osteitis) in advanced disease o Osteolysis of cortical plates in advanced disease o Pathologic fracture in advanced disease ▪ Maxillary sinus involvement: opacification, blurring of lower sinus border

**Table 3:** Imaging findings associated with BMA and MRONJ — 3D techniques

Imaging modality	Imaging features that may be observed
CBCT [11, 12]	Better view compared to imaging features observed in plain radiograph: • 3D view of a bony lesion • Osteosclerosis with confluent cortical and cancellous bone • Sequestrum formation and bone fragmentation • Periosteal reaction of solid type or multilayered onion skin appearance • Thickening of the cortical borders and maxillary sinus wall • Mandibular fracture or breach of the maxillary sinus wall in advanced disease
CT [11, 13]	In addition to imaging features observed in CBCT: • Soft tissue swelling/thickening • Maxillary sinus involvement o Mucosal thickening o Air-fluid levels o Fistula formation (oroantral or oronasal) • Cervical lymphadenopathy
MRI [11]	Findings in soft tissues may indirectly outline findings in hard tissues: • Bone marrow edema as an early sign (low T1 signal, high T2 signal) • Areas of inflammation in adjacent soft tissues (high T1 with gadolinium enhancement) • Periphery of the necrotic bone (high T1 with gadolinium enhancement, high T2) • Center of the necrotic bone (low T1 signal, low T2 signal)

*3D* Ttri-dimensional, *CBCT* cone-beam computed tomography, *CT* computed tomography, multi-detector, *MRI* magnetic resonance imaging
- General points for acceptable practice:
  - ○ There are no established pathognomonic imaging features for MRONJ, yet each imaging modality has characteristic MRONJ features.
  - ○ Plain radiographs are commonly used for initial assessment and during follow-up.
  - ○ Panoramic X-rays are valuable when MRONJ extends over a larger area than can be evaluated using periapical (PA) X-rays. It demonstrates the extent of the MRONJ and possible periosteal reaction or maxillary sinus tissue reaction.
  - ○ Cone-beam computed tomography (CBCT) provides superior detectability of the extent of bone involvement. CBCT enables determination of the field of view based on the MRONJ-affected area, which may reduce the radiation dose. Additionally, CBCT enables increased resolution, which improves the image quality, but increases the radiation dose and the likelihood of having metallic and motion artifacts.
  - ○ CT and magnetic resonance imaging (MRI) provide better information about surrounding soft tissue, muscles, neurovascular bundles, lymph nodes, and maxillary sinus. MRI may provide a window to the bone marrow. The literature suggests a higher sensitivity of MRONJ detection in CT and MRI (96% and 92%, respectively) relative to panoramic radiographs (54%) [14]. CT may be dramatically affected by “dental” artifact, which reduces its value for MRONJ assessment.
  - ○ Nuclear medicine techniques, such as scintigraphy and positron emission tomography-CT (PET-CT), can demonstrate uptake in jawbone lesions, which indicate increased bone activity. However, these techniques are not currently indicated as standard diagnostic tests for MRONJ.
  - ○ Often, cancer patients undergo routine CT, MRI, and nuclear imaging to monitor their underlying malignant disease. These imaging modalities may help in the diagnosis and monitoring of jaw pathology without prescribing additional MRONJ-directed imaging.
- Economic considerations and availability of imaging machines may drive decisions in the selection of the imaging technique. As there is great variability between countries and health insurance programs, this aspect is not covered in this CPS.

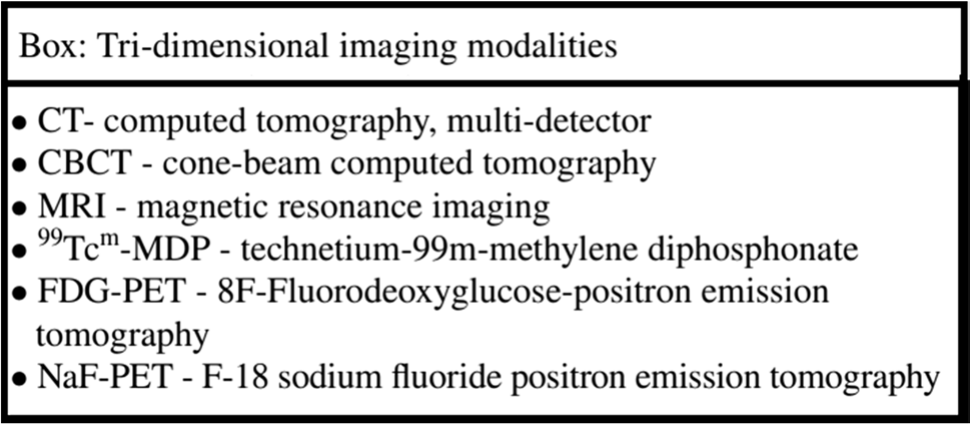

### Imaging—differential diagnosis

- Imaging tests play an important role during the diagnostic process and may help differentiate MRONJ from other pathologies.
- A PA radiograph may help rule out pathosis from adjacent dental or bony structures.
  - ○ A PA view may identify a periapical rarefication (granuloma or cyst), vertically fractured root, impacted tooth, residual root, or periodontal disease that may explain the local symptoms.
  - ○ In the presence of a fistula, a PA view using a tracer (e.g., gutta-percha) through the fistula may help detect the source of infection.
- Panoramic radiographs may identify an incidental finding of abnormal alveolar trabeculation without a diagnostic clinical presentation of MRONJ. The differential diagnosis of bone metastasis versus MRONJ should be considered. A bone biopsy would provide a definitive diagnosis. Nevertheless, it poses a risk for MRONJ development or its worsening and should be discussed with the patient and the patient’s oncologist. Radiologic follow-up is advised to assess the behavior of the bony lesion over time. If symptoms develop, empiric antibiotics may assist in the differential diagnosis.
- Like plain radiology, at present, 3D imaging, such as CBCT, CT, and MRI, is often unable to differentiate between MRONJ and bone metastasis.
- Bone scans (e.g., conventional scintigraphy, SPECT or SPECT/CT) and most types of PET-CT (e.g., FDG-PET/CT, NaF-PET/CT) show bone activity but are not specific imaging modalities for MRONJ since they demonstrate increased uptake in both inflammatory process as well as metastasis [15, 16].
- Recent technological advancements suggest that PET-CT with new tracers, such as ligands that bind to prostate-specific membrane antigen (PSMA), may be able to differentiate prostate cancer metastasis from osteonecrosis. In this technique, gallium-68 (68 Ga)-labelled PSMA ligands have higher avidity to metastasis compared to inflammatory processes in the jawbone [17]. As PSMA is expressed in solid cancers other than prostate [18], the potential of PSMA-PET/CT techniques to identify metastatic disease may have implications for MRONJ diagnosis in other solid cancer patients.
- In some PET-CT imaging, the normal uptake in the salivary glands may obscure mandibular pathology [19]. The clinician is advised to cross-check the imaging in various planes.
- Combined use of imaging may aid in the differential diagnosis of MRONJ in patients with multiple myeloma: Tc-99 m-sestamibi shows no uptake and FDG-PET/CT shows focal uptake in MRONJ [20] .

### Imaging—staging

- Staging of MRONJ is important for treatment decisions and for communication between clinicians.
- While stages 1 and 2 are determined based on the clinical findings, imaging is essential to classify MRONJ as stage 0 and stage 3 of the AAOMS scale:
  - ○ MRONJ stage 0 is diagnosed when there are asymptomatic non-specific clinical signs, symptoms of local inflammation, or symptoms without evidence of necrotic bone, in a patient with a history of BMA use. Radiologic findings such as an irregular pattern of bone trabeculation with foci of sclerosing or rarefaction, or thickening of the lamina dura, may support a diagnosis of stage 0 MRONJ.
  - ○ MRONJ stage 3 is diagnosed when the MRONJ extends beyond the alveolar bone, presents as a pathologic fracture, manifests with an extraoral fistula, or involves an oro-antral/nasal communication. Imaging may identify these features and upgrade the diagnosis to stage 3.

### Imaging—monitoring

- As part of the healing process, the necrotic bone may gradually separate from the normal surrounding alveolar bone and eventually exfoliate.
- Periodic radiographs may enable assessment of the involved site for early signs of sequestrum formation, such as the presence of well-demarcated borders of necrotic bone. The rearrangement of bone next to the sequestrum can often be observed on radiographs before there is clinical evidence of a detached sequestrum.
- The selection of the specific imaging technique is determined on a case-by-case basis depending on the advantages and disadvantages of each technique (Table 1), while attempting to use the least radiation exposure.
- If deterioration is suspected based on the clinical presentation, radiographs may confirm worsening of MRONJ by demonstrating extended involvement of bone and surrounding structures. Extension of the area of necrosis may be seen on imaging and not be appreciated on examination alone.

### Imaging—treatment decisions

- Imaging is a critical tool in preparation for maxillofacial surgery with CT and CBCT being used routinely to plan the resection and reconstruction.
- Imaging may demonstrate proximity of the MRONJ to anatomical structures that carry a high risk for post-surgical complications such as post-operative neuralgia, neuropathy, or oro-antral/nasal communication. In such cases, the radiograph may guide a conservative treatment approach.
- 3D imaging may confirm a detached sequestrum which suggests feasibility for sequestrum removal. Likewise, 3D imaging may assist in estimating the risk for post-surgery fracture.
- CT studies showing signs of periosteal reaction were correlated with lower rates of MRONJ healing, and therefore may assist with treatment decisions [21].

### Imaging—prediction

- Panoramic radiographs and CBCT were suggested to predict the development of MRONJ following a dentoalveolar procedure. Sclerosis of the trabecular bone, thickening of the lamina dura, or widening of the periodontal ligament were more frequently observed in patients who developed MRONJ eventually [22, 23]. Yet, clinicians should be careful with interpreting imaging studies given the known artifacts of panoramic radiographs and CBCT.

### Laboratory workup—diagnosis

- Considering that the diagnosis is made based primarily on clinical presentation, the role of lab workup is limited.
- It is important to review the patient’s complete blood count to confirm that the patient is not neutropenic or thrombocytopenic prior to penetrating or manipulating the soft tissue. This is clinically relevant if the MRONJ diagnosis is based on palpation of bone through a fistula, using a hand instrument (periodontal probe). If the neutrophil or platelet count demonstrates that the patient is at risk of infection or bleeding, respectively, the clinical examination should be adjusted accordingly.

### Laboratory workup—differential diagnosis

- Certain infectious diseases may share clinical features with MRONJ, such as osteomyelitis or bone exposure. Therefore, based on the clinical circumstances and review of systems, other infections should be considered. For example, deep fungal infection (e.g., aspergillosis, mucormycosis), viral infection (e.g., varicella zoster virus reactivation), or bacterial infection (e.g., tuberculosis). In such cases, laboratory workup is driven by the differential diagnosis.

### Laboratory workup—monitoring

- Elevated C-reactive protein (CRP) value indicates inflammation, although the specificity of this test in monitoring MRONJ is low. This test is commonly used in oncologic patients and may be readily available for the clinicians who manage MRONJ. Of note, the literature reports higher CRP levels to correlate with the severity of acute inflammation and larger osteolysis size on a light microscopy study [24, 25].
- Uncontrolled diabetes is associated with delayed wound healing. Therefore, in diabetic MRONJ patients, HbA1C level should be checked over time as it indicates glycemic control.
- Reduction in the white blood cell count (WBC) was reported to be associated with the recurrence of MRONJ [26]. Therefore, WBC should be monitored, and if values are low, patients should be followed up closely.
- MRONJ-related sepsis is a rare complication that may develop in patients with multiple risk factors. It is an emergency that needs to be monitored and managed according to the standard workflow for sepsis.

### Laboratory workup—treatment decisions

- Leukopenia is common in oncologic patients, either due to the cancer itself or the anti-cancer therapy, which increases the risk of secondary infection of MRONJ lesions or the spread of infection. Leukopenia may be one of the considerations favoring a conservative approach such as antibiotic treatment for MRONJ as opposed to a surgical approach. Additionally, for neutropenic patients who are treated with antibiotics for MRONJ, a longer-than-usual treatment duration may be needed.
- If dentoalveolar surgery is deemed necessary in a severe neutropenic MRONJ patient, antibiotic prophylaxis should be considered and coordinated with the oncology team.
- Bleeding tendencies may arise due to thrombocytopenia or coagulation disorders which are common in cancer patients. Furthermore, cancer-related thrombosis may require anticoagulation therapy. A complete blood count and/or coagulation test to determine the risk of bleeding should be considered before a dentoalveolar procedure.
- Additional risk factors for bleeding, such as blood wall fragility (e.g., in multiple myeloma) and medication-related adverse effects (e.g., bevacizumab), may exist. The clinician should be aware that thrombocytopenia and abnormal coagulation tests may not be the only indication for a higher risk of bleeding.
- Some serologic bone turnover markers were suggested to be associated with faster MRONJ healing (higher CTX, higher osteocalcin, lower 1,25-dihydroxyvitamin D, and higher bone-specific alkaline phosphatase) [27]. However, due to the limited evidence, the validity of these results and their practical implications remain unknown.
- Lower CRP level has been reported to correlate with better post-surgical healing [24]. This inflammatory marker is commonly used in cancer patients; however, it is influenced by numerous factors which reduce its value for MRONJ treatment decisions.

### Laboratory workup—prediction

- Ideally, predictive laboratory tests, such as bone turnover markers and genetic markers, may help with stratifying the risk for MRONJ, which in turn may help to determine whether to proceed with surgery and when, but there is insufficient evidence for current clinical application.
- Since bone turnover markers may change over time, studies intended to assess the use of these tests as predictors for MRONJ are meaningful only if tested at the time of the tooth extraction in a prospective manner. Secondly, the sample size should have sufficient power to reflect the prevalence of MRONJ per the patient population (cancer versus osteoporosis). Readers are advised to carefully interpret the results of clinical trials or of systematic reviews that include mixed methodology studies in respect to the study design described above.
- The bone turnover marker C-terminal telopeptide (CTX) has been reported to predict MRONJ development. CTX values lower than 150 pg/mL reportedly correlate with a higher risk for MRONJ development in patients treated with bisphosphonates [28, 29].
- There is no evidence for the potential of CTX as a MRONJ predictive tool in patients treated with denosumab.
- The search for predictive tools continues, including research on serologic tests, salivary proteomics, and biogenetic markers. The translation of these findings into practical tools is challenging and relies on the commercialization of these tests.

## Supplementary Information

Below is the link to the electronic supplementary material.

ESM 1 (DOCX 17.4 KB)

## Data Availability

No datasets were generated or analysed during the current study.

## Declarations

### Disclaimer

The MASCC/ISOO OCSG Statements have been developed to facilitate expert-opinion-based management of oral complications of cancer and cancer therapy where high-quality evidence is lacking. Clinicians should use their judgment when making treatment decisions for individual patients. Statement authors and the MASCC/ISOO do not guarantee or take responsibility for the clinical outcomes in individual patients.
