# Supplementary material for: MASCC/ISOO Clinical Practice Statement: imaging and clinical laboratory tests in the diagnosis and management of medication-related osteonecrosis of the jaw
Source: Support Care Cancer. 2025 Sep 13;33(10):852. doi: 10.1007/s00520-025-09809-8 (PMC12433361; doi:10.1007/s00520-025-09809-8)
Supplement: Supplementary file 1 — (DOCX 17.4 KB) [file 520_2025_9809_MOESM1_ESM.docx]

**Supplementary information**

**MASCC/ISOO Clinical Practice Statement: Imaging and Clinical Laboratory Tests in The Diagnosis and Management of Medication Related Osteonecrosis of the Jaw**

**Suggested reading**

1. Kim JE, Yoo S, Choi SC. Several issues regarding the diagnostic imaging of medication-related osteonecrosis of the jaw. Imaging Sci Dent. 2020 Dec;50(4):273-279. doi: 10.5624/isd.2020.50.4.273. Epub 2020 Dec 15. PMID: 33409135; PMCID: PMC7758260.

1. Campisi G, Mauceri R, Bertoldo F, Bettini G, Biasotto M, Colella G, Consolo U, Di Fede O, Favia G, Fusco V, Gabriele M, Lo Casto A, Lo Muzio L, Marcianò A, Mascitti M, Meleti M, Mignogna MD, Oteri G, Panzarella V, Romeo U, Santarelli A, Vescovi P, Marchetti C, Bedogni A. Medication-Related Osteonecrosis of Jaws (MRONJ) Prevention and Diagnosis: Italian Consensus Update 2020. Int J Environ Res Public Health. 2020 Aug 18;17(16):5998. doi: 10.3390/ijerph17165998. PMID: 32824826; PMCID: PMC7460511.
2. Fanti S, Goffin K, Hadaschik BA, Herrmann K, Maurer T, MacLennan S, Oprea-Lager DE, Oyen WJ, Rouvière O, Mottet N, Bjartell A. Consensus statements on PSMA PET/CT response assessment criteria in prostate cancer. Eur J Nucl Med Mol Imaging. 2021 Feb;48(2):469-476. doi: 10.1007/s00259-020-04934-4. Epub 2020 Jul 2. PMID: 32617640; PMCID: PMC7835167.
3. Elad S, Gomori MJ, Ben-Ami N, Friedlander-Barenboim S, Regev E, Lazarovici TS, Yarom N. Bisphosphonate-related osteonecrosis of the jaw: clinical correlations with computerized tomography presentation. Clin Oral Investig. 2010 Feb;14(1):43-50. doi: 10.1007/s00784-009-0311-3. Epub 2009 Jul 15. PMID: 19603201.
4. Cochrane Database Syst Rev . 2022 Jul 12;7(7):CD012432. doi: 10.1002/14651858.CD012432.pub3. Interventions for managing medication-related osteonecrosis of the jaw Natalie H Beth-Tasdogan 1, Benjamin Mayer 2, Heba Hussein 3, Oliver Zolk 4, Jens-Uwe Peter 4 PMID: 35866376
5. Nashi M, Hirai T, Iwamoto T, Takenobu T. Clinical risk factors for severity and prognosis of antiresorptive agent-related osteonecrosis of the jaw: a retrospective observational study. J Bone Miner Metab. 2022 Nov;40(6):1014-1020. doi: 10.1007/s00774-022-01367-x. Epub 2022 Sep 27. PMID: 36166107.
6. [Yui Yin Ko](https://pubmed-ncbi-nlm-nih-gov.ezlibrary.technion.ac.il/?term=Ko+YY&cauthor_id=39202187) , [Wei-Fa Yang](https://pubmed-ncbi-nlm-nih-gov.ezlibrary.technion.ac.il/?term=Yang+WF&cauthor_id=39202187) , [Yiu Yan Leung](https://pubmed-ncbi-nlm-nih-gov.ezlibrary.technion.ac.il/?term=Leung+YY&cauthor_id=39202187) . The Role of Cone Beam Computed Tomography (CBCT) in the Diagnosis and Clinical Management of Medication-Related Osteonecrosis of the Jaw (MRONJ). Diagnostics (Basel) 2024 Aug 6;14(16):1700. doi: 10.3390/diagnostics14161700. PMID: 39202187
7. [Amanda Katarinny Goes Gonzaga](https://pubmed-ncbi-nlm-nih-gov.ezlibrary.technion.ac.il/?term=Gonzaga+AKG&cauthor_id=39371309) , [Hannah Gil de Farias Morais](https://pubmed-ncbi-nlm-nih-gov.ezlibrary.technion.ac.il/?term=Morais+HGF&cauthor_id=39371309), [Camila Dayla Melo Oliveira](https://pubmed-ncbi-nlm-nih-gov.ezlibrary.technion.ac.il/?term=Oliveira+CDM&cauthor_id=39371309) , [Magda Lyce Rodrigues Campos](https://pubmed-ncbi-nlm-nih-gov.ezlibrary.technion.ac.il/?term=Campos+MLR&cauthor_id=39371309)[3](https://pubmed-ncbi-nlm-nih-gov.ezlibrary.technion.ac.il/39371309/#full-view-affiliation-3), [Carolina Raiane Leite Dourado Maranhão Diaz](https://pubmed-ncbi-nlm-nih-gov.ezlibrary.technion.ac.il/?term=Diaz+CRLDM&cauthor_id=39371309) , [Marcos Custódio](https://pubmed-ncbi-nlm-nih-gov.ezlibrary.technion.ac.il/?term=Cust%C3%B3dio+M&cauthor_id=39371309), [Natália Silva Andrade](https://pubmed-ncbi-nlm-nih-gov.ezlibrary.technion.ac.il/?term=Andrade+NS&cauthor_id=39371309) , [Thalita Santana](https://pubmed-ncbi-nlm-nih-gov.ezlibrary.technion.ac.il/?term=Santana+T&cauthor_id=39371309) . Imaging aspects of maxillomandibular bone alterations in patients with multiple myeloma treated with bisphosphonates: A systematic review. Imaging Sci Dent. 2024 Sep;54(3):221-231. doi: 10.5624/isd.20240032. Epub 2024 Jul 17. PMID: 39371309
8. [Elif Aslan](https://pubmed-ncbi-nlm-nih-gov.ezlibrary.technion.ac.il/?term=Aslan+E&cauthor_id=38810135) , [Erinc Onem](https://pubmed-ncbi-nlm-nih-gov.ezlibrary.technion.ac.il/?term=Onem+E&cauthor_id=38810135), [Ali Mert](https://pubmed-ncbi-nlm-nih-gov.ezlibrary.technion.ac.il/?term=Mert+A&cauthor_id=38810135) , [B Guniz Baksi](https://pubmed-ncbi-nlm-nih-gov.ezlibrary.technion.ac.il/?term=Baksi+BG&cauthor_id=38810135) . Comparison of quantitative radiomorphometric predictors of healthy and MRONJ-affected bone using panoramic radiography and cone-beam CT. Dentomaxillofac Radiol . 2024 Sep 1;53(6):407-416 doi: 10.1093/dmfr/twae024. PMID: 38810135
9. Abdolrahmani A, Epstein JB, Samim F. Medication-related osteonecrosis of the jaw: evolving research for multimodality medical management. Support Care Cancer. 2024 Mar 5;32(4):212. doi: 10.1007/s00520-024-08388-4. PMID: 38443685.
